# Epigenetic program and transcription factor circuitry of dendritic cell development

**DOI:** 10.1093/nar/gkv1056

**Published:** 2015-10-17

**Authors:** Qiong Lin, Heike Chauvistré, Ivan G. Costa, Eduardo G. Gusmao, Saskia Mitzka, Sonja Hänzelmann, Bianka Baying, Theresa Klisch, Richard Moriggl, Benoit Hennuy, Hubert Smeets, Kurt Hoffmann, Vladimir Benes, Kristin Seré, Martin Zenke

**Affiliations:** 1 Institute for Biomedical Engineering, Department of Cell Biology, RWTH Aachen University Medical School, 52074 Aachen, Germany; 2 Helmholtz Institute for Biomedical Engineering, RWTH Aachen University, 52074 Aachen, Germany; 3 Department of Dermatology, University Hospital Essen, 45147 Essen, Germany; 4 IZKF Computational Biology Research Group, RWTH Aachen University Medical School, 52074 Aachen, Germany; 5 Aachen Institute for Advanced Study in Computational Engineering Science (AICES), RWTH Aachen University, 52062 Aachen, Germany; 6 Genomics Core Facilities GeneCore, European Molecular Biology Laboratory (EMBL), 69117 Heidelberg, Germany; 7 Ludwig Boltzmann Institute for Cancer Research, University of Veterinary Medicine, Medical University Vienna, 1090 Vienna, Austria; 8 GIGA-Genomics, University of Liège, 4000 Liège, Belgium; 9 Department of Genetics and Cell Biology, Maastricht University Medical Center, 6200 MD Maastricht, The Netherlands; 10 Research Schools CARIM and GROW, Maastricht University Medical Center, 6200 MD Maastricht, The Netherlands; 11 Institute of Molecular Biotechnology, RWTH Aachen University, 52074 Aachen, Germany

## Abstract

Dendritic cells (DC) are professional antigen presenting cells that develop from hematopoietic stem cells through successive steps of lineage commitment and differentiation. Multipotent progenitors (MPP) are committed to DC restricted common DC progenitors (CDP), which differentiate into specific DC subsets, classical DC (cDC) and plasmacytoid DC (pDC). To determine epigenetic states and regulatory circuitries during DC differentiation, we measured consecutive changes of genome-wide gene expression, histone modification and transcription factor occupancy during the sequel MPP-CDP-cDC/pDC. Specific histone marks in CDP reveal a DC-primed epigenetic signature, which is maintained and reinforced during DC differentiation. Epigenetic marks and transcription factor PU.1 occupancy increasingly coincide upon DC differentiation. By integrating PU.1 occupancy and gene expression we devised a transcription factor regulatory circuitry for DC commitment and subset specification. The circuitry provides the transcription factor hierarchy that drives the sequel MPP-CDP-cDC/pDC, including Irf4, Irf8, Tcf4, Spib and Stat factors. The circuitry also includes feedback loops inferred for individual or multiple factors, which stabilize distinct stages of DC development and DC subsets. In summary, here we describe the basic regulatory circuitry of transcription factors that drives DC development.

## INTRODUCTION

Dendritic cells (DC) represent specialized immune cells that develop from hematopoietic stem cells (1,2). DC are widely distributed in both lymphoid and non-lymphoid tissues and bridge innate and adaptive immune responses. DC function builds on their capacity to capture, process and present antigens to T cells (1,3,4). DC are divided into distinct subsets according to their localization, phenotype and function (1,3,4). Lymphoid tissues contain classical/conventional DC (cDC) and plasmacytoid DC (pDC), which represent the main DC subsets. Peripheral organs contain migratory tissue DC, which capture antigens and migrate to lymphoid organs for antigen presentation to T cells.

DC development from hematopoietic stem cells comprises two critical steps: DC commitment and DC subset specification (1,2,5). First, multipotent hematopoietic stem/progenitor cells (MPP) are committed toward the DC lineage, which yields the DC-restricted common DC progenitor (CDP). Second, CDP further develop into the specific DC subsets, cDC and pDC. cDC are specialized for antigen processing and presenting, while pDC produce large amounts of type I interferon e.g. in response to viral infections.

Genome-wide gene expression and gene knockout studies in mice identified several critical regulators for DC commitment and subset specification, such as Flt3, Stat3, Id2, Irf8 and Tcf4 (1,3,6–13). Hematopoietic master regulators, such as the transcription factors PU.1 and Gfi1, were also shown to regulate DC development (3,6,14,15). However, how the various transcription factors interact to regulate DC development has remained elusive.

Epigenetic mechanisms regulate cell development, identity and function. This occurs by positioning specific histone modifications at promoter and enhancer sequences that impact on transcription factor binding and thus gene expression (16,17). Histone H3 lysine 4 trimethylation (H3K4me3) and H3 lysine 27 trimethylation (H3K27me3) at gene promoters are associated with gene activation and repression, respectively. H3 lysine 4 monomethylation (H3K4me1) marks genomic regions that indicate primed enhancers. Additionally, key developmental genes have bivalent modification where large domains of repressive H3K27me3 coexist with small domains of activating H3K4me3 (18–21). These genes are poised/primed for either activation or repression during differentiation. Chromatin structure and transcription factor binding provide the foundation for the topology of complex gene regulatory networks that determine cell fate decisions (16,17,22).

Epigenetic modifications and transcription factors also regulate hematopoiesis, the development of hematopoietic stem cells into all cells in blood and blood-borne lymphoid organs (22,23). Hence, current efforts on high-throughput mapping of histone modifications and transcription factor binding are directed toward elucidating the regulatory codes that drive lineage commitment and differentiation during hematopoiesis (23–26). For example, specific histone modification patterns control hematopoietic stem cells, T cell development and erythropoiesis (20,27,28). Global histone modification and transcription factor occupancy in inflammatory DC stimulated with lipopolysaccharide and in monocyte-derived DC and pDC were also studied (26,29). Recent genomic studies on blood cell formation from hematopoietic stem cells covered all conventional hematopoietic lineages, but did not include DC (23).

Here, we determined how DC transcription factors are wired to drive DC lineage commitment and subset specification. First, we generated high resolution genome-wide maps of gene expression, histone modification and transcription factor occupancy in MPP, CDP, cDC and pDC. Second, we developed an integrative computational approach by combining differential transcription factor binding, gene expression data and motif enrichment analysis to reverse engineer a DC regulatory circuitry for DC commitment and subset specification. The circuitry was further validated and provides the transcription factor hierarchy that drives the sequel MPP-CDP-cDC/pDC and includes several feedback loops that stabilize distinct stages of DC development and DC subsets.

## MATERIALS AND METHODS

### Cell culture

Culture of progenitor cells from mouse bone marrow and their differentiation into DC were done as previously described (5). MPP, CDP, cDC and pDC were obtained by FACS sorting (FACSAria, BD Biosciences) and used for RNA preparation and chromatin immunoprecipitation (ChIP).

Bone marrow cells of Irf8+/+ and Irf8−/− mice (30) were cultured as described in Felker *et al*. (5) and CDP (Gr1-Flt3+c-kit+M-CSFR+) were obtained by FACS sorting. RNA was prepared (RNeasy Mini Kit with DNase I digestion, Qiagen) and subjected to gene expression profiling (Affymetrix Mouse Gene 1.0 ST Array) as described (5). DC progenitors (5) were infected with Stat5a-ER-GFP retrovirus vector (31) containing a constitutively active tamoxifen (tmx) inducible Stat5. Cells were stimulated with tmx for 8, 16 and 24 hours and GFP+ and GFP- cells were isolated by FACS sorting. Samples were subjected to gene expression profiling as above.

### Gene expression analysis

DNA microarray data are from GSE22432, GSE15907, GSE34915 and GSE45467 and were analyzed as described (5). Differentially expressed genes between two cell types were detected using limma *t*-test with criteria of fold change >2 and *P* values < 0.05. Raw *P* values were adjusted by Benjamini–Hochberg multiple test correction (32). To generate lineage-specific clusters, all differentially expressed genes were subjected to fuzzy *c*-mean algorithm (33) and further aggregated into six clusters according to their gene expression patterns. Clusters were depicted in heat map format. The boxplot analysis of gene expression data was done in R. Gene set enrichment analysis was performed using DAVID bioinformatics tools (34).

### Chromatin immunoprecipitation and deep sequencing (ChIP-seq) analysis

ChIP assays were performed as described (35) with minor modifications (see Supplementary Data). The ChIP-seq data are available from NCBI GEO series GSE57563 and GSE64767 and visualized by a customized UCSC genome browser track data hub (http://www.molcell.rwth-aachen.de/dc/). The PU.1 ChIP-seq data are from GSE21953, GSE31233, GSE21621 and GSE36104. The ChIP-seq data of Irf4, Irf8 and Stat3 in cDC and Irf8 and Tcf4 in pDC are from GSE36104, GSE53311, GSE27161, GSE62702, GSE43876 and GSE66899 (see also CODEX database (36)).

Short reads of the ChIP-seq experiments were aligned to the mouse reference genome (NCBI37/mm9) using Bowtie. The aligned ChIP-seq data sets of transcription factor PU.1 and the enhancer mark H3K4me1 were then processed to identify genomic regions with sequence enrichment described as peaks using MACS software (version 1.4.2; (37)). Peaks of H3K4me3 and H3K27me3 were identified using SICER, a spatial clustering approach for the identification of larger ChIP-enriched regions (38). The signals of specific histone modifications (H3K4me3 and H3K27me3) for gene centric analysis were obtained by calculating the average tag density in ±1 kb interval centered around transcription start site (TSS) for each Refseq annotated gene. The signals of PU.1 and the enhancer mark H3K4me1 were calculated by the average tag density of ±50 kb centered around TSS. Principal component analysis (PCA) was based on the PU.1 peaks identified in MPP, CDP, cDC and pDC. The peak signals for these PU.1 data sets (GSE57563) and reference PU.1 data sets (GSE21953, GSE31233, GSE21621 and GSE36104) were calculated by the coverage of reads within the peaks. Data were quantile normalized and subjected to PCA.

### Identification of PU.1 co-binding transcription factors and construction of DC regulatory networks

An integrative approach was designed to detect transcription factor motifs that are highly enriched in PU.1 peaks. Briefly, we focused on differentially expressed transcription factors upon DC development and collected their sequence motifs from public databases. We then performed motif search around PU.1 differential peaks to determine the enrichment of transcription factor binding sites for each cell type and to identify cell-specific PU.1 co-binding partners. PU.1 co-binding transcription factors and selected key DC regulators from the literature (6,39–45) were used to build lineage-specific transcription factor networks and the DC regulatory circuitry (see details in Supplementary Data). A further enrichment analysis was performed on footprints of cell-specific H3K4me1 peaks following Gusmao *et al*. (46). This general workflow is based on tools from the Regulatory Genomics toolbox (www.regulatory-genomics.org).

## RESULTS

### Global maps of gene expression and histone modification in DC development

**Figure 1.**
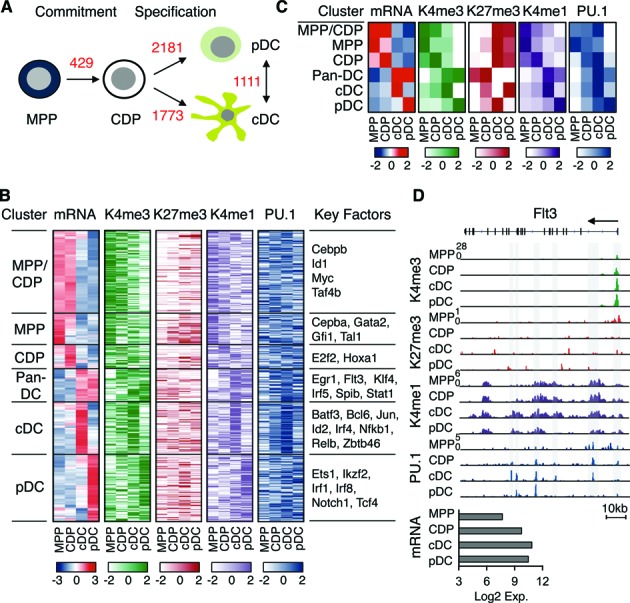
Gene expression, H3K4me1, H3K4me3, H3K27me3 and PU.1 occupancy in DC development. (**A**) Schematic representation of DC commitment from MPP to CDP and DC subset specification from CDP to cDC or pDC. The number of differentially expressed genes between a pair of cell states is given in red. (**B**) Differentially expressed genes were clustered according to their expression in MPP/CDP, MPP, CDP, pan-DC, cDC and pDC as indicated. Heat map representation of gene specific mRNA expression (red, high expression; blue, low expression) and the respective H3K4me1, H3K4me3, H3K27me3 and PU.1 occupancy is shown (dark colors indicate high occupancy; light or white colors indicate low or no occupancy). H3K4me3 and H3K27me3 signals at promoter regions (TSS±1kb); H3K4me1 and PU.1 signals at distal regions/enhancers (TSS±50kb). Key regulatory factors are listed. (**C**) For each of the six clusters, mRNA expression, H3K4me1, H3K4me3, H3K27me3 and PU.1 occupancy were calculated using the geometric mean of the levels of respective genes and are shown in heat map format. Color code as in (B). (**D**) Occupancy for H3K4me1, H3K4me3, H3K27me3, PU.1 and mRNA profile (log2 expression) of Flt3 gene in MPP, CDP, cDC and pDC. Promoter and enhancer regions with PU.1 binding are indicated (gray box).

MPP were induced to differentiate *in vitro* into CDP (DC commitment) and further into cDC and pDC (DC subset specification; Figure 1A; (5,47)). A total of 3194 genes were differentially expressed between the differentiation stages (fold change > 2, *P* value < 0.05). An increasing number of genes was found to be differentially regulated during DC commitment (429 genes) and DC subset specification (1773 and 2181 genes; Figure 1A). Among them, 210 genes encode transcription factors including many critical DC regulators, such as Irf4, Irf8, Batf3, Relb, Id2, Spib and Tcf4 (also known as E2–2; (3)). Differentially expressed genes were categorized in six cell-specific clusters: progenitors (MPP/CDP), MPP, CDP, Pan-DC (cDC/pDC), cDC and pDC cluster (Figure 1B and Supplementary Table S1). These clusters match very well to gene expression profiles calculated from *in vivo* sorted DC progenitors and DC (Supplementary Figure S1; (11)) and functional enrichment of GO terms (Supplementary Table S1).

The MPP cluster contains Gata2, Gfi1 and Tal1, which are down-regulated from MPP to CDP and have low or no expression in DC. This reflects the gradual restriction of development from early hematopoietic progenitors toward DC committed progenitors and DC (Figure 1B). The pan-DC cluster comprises genes, such as Flt3, with important roles in both cDC and pDC (Figure 1B). The cDC and pDC clusters identify DC subset-specific genes, such as Id2, Irf4 and Zbtb46 for cDC and Irf1 and Tcf4 for pDC. Taken together, our analysis captures known and putative DC regulators and thus provides the basis for investigating the underlying epigenetic architecture and transcriptional circuitry of DC commitment and subset specification.

Next we determined how histone marks (H3K4me1, H3K4me3 and H3K27me3) and PU.1 binding relate to stage-specific gene clusters in DC development by ChIP-seq for MPP, CDP, cDC and pDC (Figure 1B and C). The active mark H3K4me3 in MPP and CDP was confined to progenitor genes, while H3K27me3 was observed for these genes in cDC and pDC. Conversely, H3K4me3 in cDC and pDC was observed for DC genes, while H3K27me3 was seen for these genes in progenitors (Figure 1B and C). Importantly, the enhancer mark H3K4me1 and PU.1 binding were detected in both progenitor and DC specific genes and followed the pattern of H3K4me3. Moreover, PU.1 occupancy was observed for all stage-specific gene clusters in CDP, suggesting that CDP acquire a DC-primed PU.1 binding profile during DC commitment. This observation is also in line with the role of PU.1 as a pioneer transcription factor in cell fate specification (25,48,49).

### DC-specific epigenetic priming in CDP

To investigate DC epigenetic priming in CDP, we focused on genes differentially expressed during DC commitment (Figure 1A). Upon MPP-CDP transition, up-regulated genes acquire H3K4me1, H3K4me3 and PU.1 in CDP, whereas down-regulated genes acquire H3K27me3 or bivalent marks without obvious change in PU.1 occupancy (Supplementary Figure S2A and B). Importantly, these patterns are maintained in differentiated DC (cDC and pDC), indicating epigenetic DC priming of lineage-specific gene promoters and enhancer regions. Additionally, genes with an increase of H3K4me3 (e.g. Cd74) during DC commitment are associated with immune related functions, such as immune response and leukocyte activation (Supplementary Figure S2C and D).

The DC-primed epigenetic signatures in CDP are enhanced upon CDP-cDC or CDP-pDC transition, which is particularly prominent for H3K4me1 and PU.1 occupancy (Supplementary Figure S3A and B). For example, the cDC gene Cd83 shows an increase in H3K4me1, H3K4me3 and PU.1 occupancy during CDP-cDC transition and is repressed in pDC due to bivalent modifications (Supplementary Figure S3C). The epigenetic profile of the pDC gene Siglec-H during CDP-pDC transition changed accordingly (Supplementary Figure S3D). Collectively, CDP acquire DC lineage-specific epigenetic signatures during DC commitment. These lineage-primed H3K4me1, H3K4me3 and PU.1 marks initiate the transcriptional program, and thus drive DC commitment and subset specification, while H3K27me3 mark restricts alternative developmental options.

**Figure 2.**
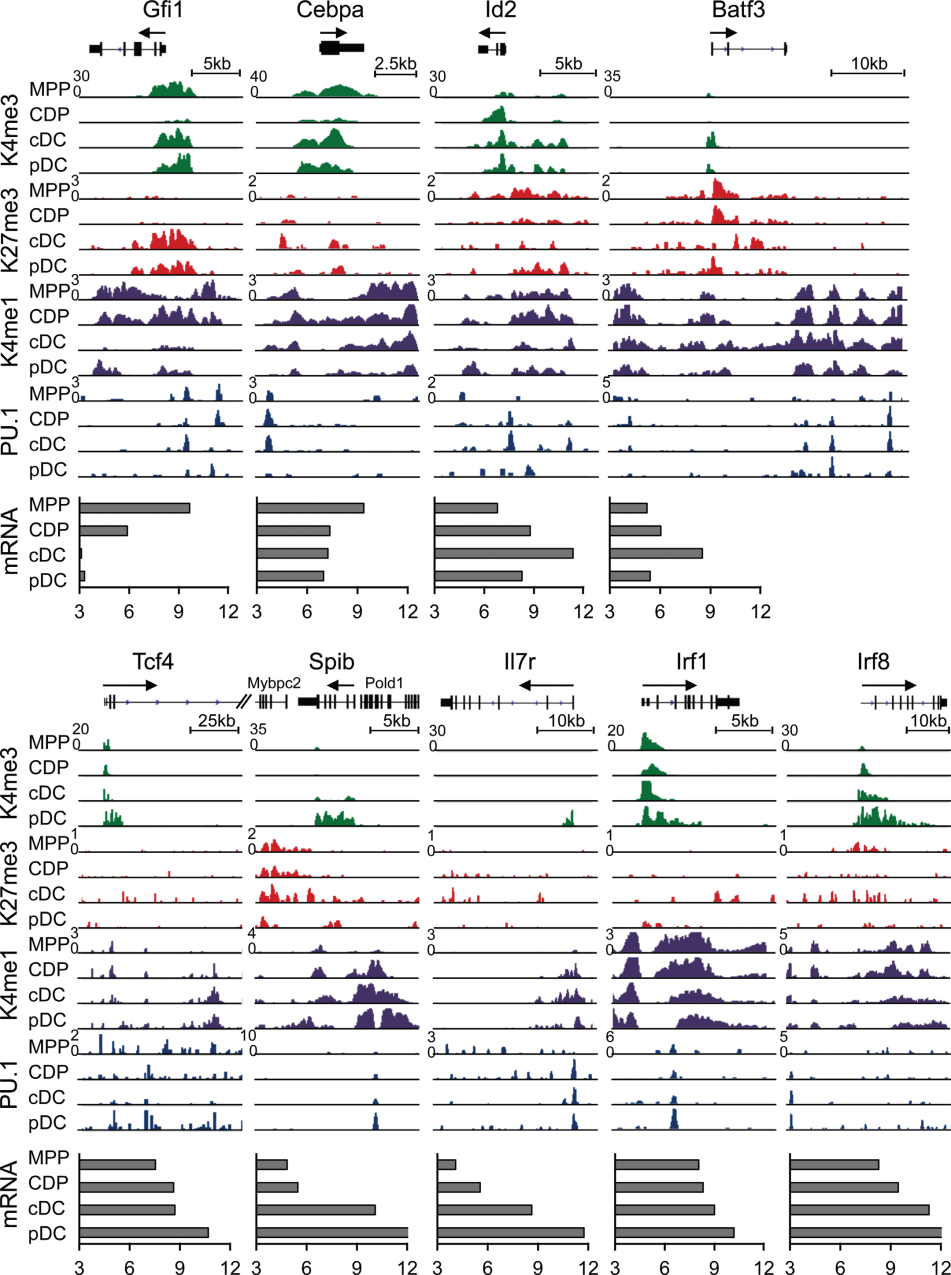
Histone modifications and PU.1 binding dynamics of key DC transcriptional regulators during DC development. Occupancy for H3K4me1, H3K4me3, H3K27me3 and PU.1 and mRNA expression (log2 expression) of DC progenitor genes (Gfi1 and Cebpa), cDC genes (Id2, Batf3) and pDC genes (Tcf4, Spib, Irf7r and Irf1) in MPP, CDP, cDC and pDC. Irf8, a central DC transcription factor, is also shown. Arrow indicates the direction of transcription.

Next, we focused on the analysis of individual representative genes, including Gfi1, Flt3, Id2 and Irf8 for progenitor, pan-DC, cDC and pDC affiliated genes, respectively (Figures 1D and 2). All these genes are implicated in DC development based on gene knockout studies (see below Supplementary Figure S9; (1,3)). Flt3 is a key cytokine receptor for DC development and regulated by PU.1 (6). Flt3 promoter shows prominent H3K4me3 signals, which increase upon differentiation, concomitantly with a decrease of H3K27me3 signals (Figure 1D). H3K4me1 and PU.1 peaks reveal multiple enhancer regions in the body of the Flt3 gene in CDP, cDC and pDC, which relate to an increase of Flt3 expression upon DC differentiation.

The progenitor gene Gfi1 has prominent H3K4me3 signals at the promoter in MPP and acquires H3K27me3 upon DC differentiation, thus forming a bivalent modification (Figure 2). A similar pattern was observed for Cepba. cDC genes (Id2, Batf3) show an accumulation of H3K4me3 and PU.1 signals in promoter or enhancer regions, respectively, in cDC. The pDC gene Spib shows pronounced H3K4me3 and PU.1 signals at the promoter in pDC. Similar chromatin profiles were also observed for the pDC genes Tcf4 and Il7r. The Irf family genes Irf1 and Irf8 show particular prominent H3K4me3 occupancy at promoters in DC, which increase during DC development (Figure 2). In summary, our analysis reveals the stage-specific changes in histone marks and PU.1 binding that are associated with DC differentiation.

### PU.1 in lineage fate determination of DC

**Figure 3.**
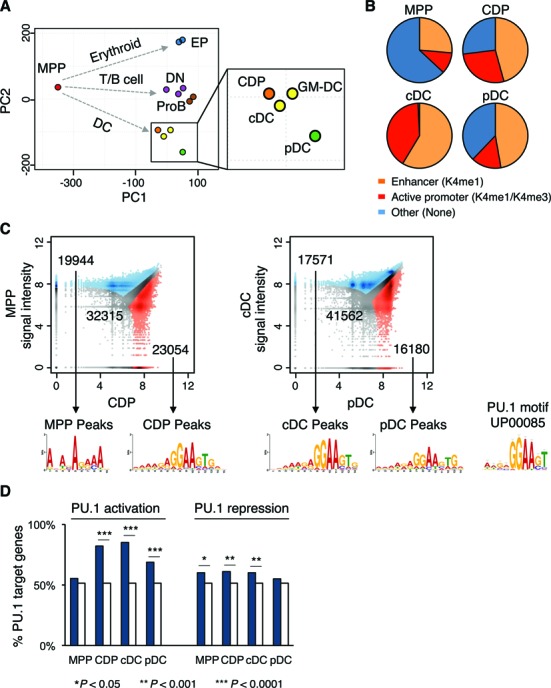
PU.1 transcription factor binding in DC development. (**A**) Principal component analysis of genome-wide PU.1 binding profiles in MPP, erythroid progenitor cells (EP), T/B lymphoid cells (double negative T cells, DN; pro B cells, ProB) and DC progenitors and subsets (CDP, cDC and pDC; GM-CSF derived DC, GM-DC). The DC cluster is highlighted. (**B**) PU.1 binding peaks occurring in enhancers or active promoters in MPP, CDP, cDC and pDC are shown. Regions were defined as active promoters if marked with both H3K4me1 and H3K4me3, and enhancers if marked only with H3K4me1. (**C**) Differential PU.1 peaks between MPP versus CDP (left) and cDC versus pDC (right) are depicted in blue and red as indicated. Non-differential peaks are colored in gray. De-novo PU.1 motifs calculated for cell type-specific peaks and the classical PU.1 motif (UP00085; (53)) are shown. (**D**) The proportion of PU.1 target genes with differential PU.1 peaks close to differentially regulated genes in progenitors (MPP versus CDP) and DC subsets (cDC versus pDC) are shown in percent (filled bars). The percentage of PU.1 targets in all genes was used as background control (open bars). The Fisher's exact test was employed to calculate the enrichment of PU.1 targets.

PU.1 (encoded by Sfpi1 gene) represents a master regulator in hematopoiesis with a prominent role in multiple cell fate decisions, including DC development (6,14). PU.1 occupancy in differentially regulated genes during DC commitment and specification is prominently up-regulated (Supplementary Figures S2A and S3A and B), suggesting that PU.1 has a determining function in establishing the DC lineage. Principal component analysis of PU.1 ChIP-seq data of DC and multiple hematopoietic lineages (26,50–52) reveals a DC-specific developmental pathway induced by PU.1 (Figure 3A). Interestingly, CDP cluster with DC and are positioned distant from MPP, indicating that CDP exhibit a DC-primed PU.1 binding profile. cDC and GM-DC are positioned very close to CDP, which supports the hypothesis that differentiation of cDC represents the default pathway of DC development (3,6,39).

Genome-wide analysis of PU.1 ChIP-seq data reveals a preference for PU.1 binding to gene bodies and intergenic regions in MPP, CDP, cDC and pDC (Supplementary Figure S4A). This is consistent with previous studies and PU.1 function as an enhancer factor (25,26). Additionally, a prominent increase of PU.1 binding is observed in the promoter regions from MPP to CDP and cDC (Supplementary Figure S4A). An increasing number of PU.1 peaks in CDP, cDC and pDC were found in active promoter and enhancer regions (H3K4me3 and H3K4me1, respectively) compared to MPP (Figure 3B). A similar trend was observed in GM-DC ChIP-seq data (Supplementary Figure S4B; (26)). These results are in line with PU.1 recruiting chromatin modifiers (25) and initiating DC lineage commitment. Moreover, the genomic distribution of PU.1 peaks is different for cDC and pDC (Supplementary Figure S4A), indicating that PU.1 is also involved in DC lineage diversification.

Next, we analyzed the consecutive changes of PU.1 peaks during DC development and the associated PU.1 motifs (Figure 3C). A total of 19 944 MPP and 23 054 CDP differential peaks were detected. MPP peaks reveal an alternative de-novo motif with a weak GGAA sequence, while CDP peaks show the classical PU.1 motif (UP00085; (53)), containing an ETS binding site (GGAA core site; Figure 3C). Similarly, PU.1 occupancy is also different between cDC and pDC. The PU.1 binding sequence in cDC resembles the classical PU.1 motif, while the pDC binding sequence has a similar motif with less specificity (Figure 3C). These results are in support of DC-primed PU.1 binding profiles in CDP. They also suggest that using different PU.1 binding characteristics represents yet another level of fine-tuning stage- and cell type-specific gene regulation. Furthermore, PU.1 binding was significantly associated with transcriptional activation of lineage-specific genes in CDP, cDC and pDC (Figure 3D), indicating a positive role of PU.1 in DC lineage fate determination.

### Identification of PU.1 co-binding transcription factors

Transcription factor networks control hematopoietic cell differentiation, including DC development (3,4,11,54,55). Genome-wide approaches, interrogating gene expression, ChIP-seq data and transcription factor binding motifs, have been used to identify gene regulatory network (26,56). To identify PU.1 co-binding partners in DC development, we designed an integrative computational approach to analyze sequences around PU.1 peaks of differentially expressed genes (see Materials and Methods; Supplementary Figure S5).

**Figure 4.**
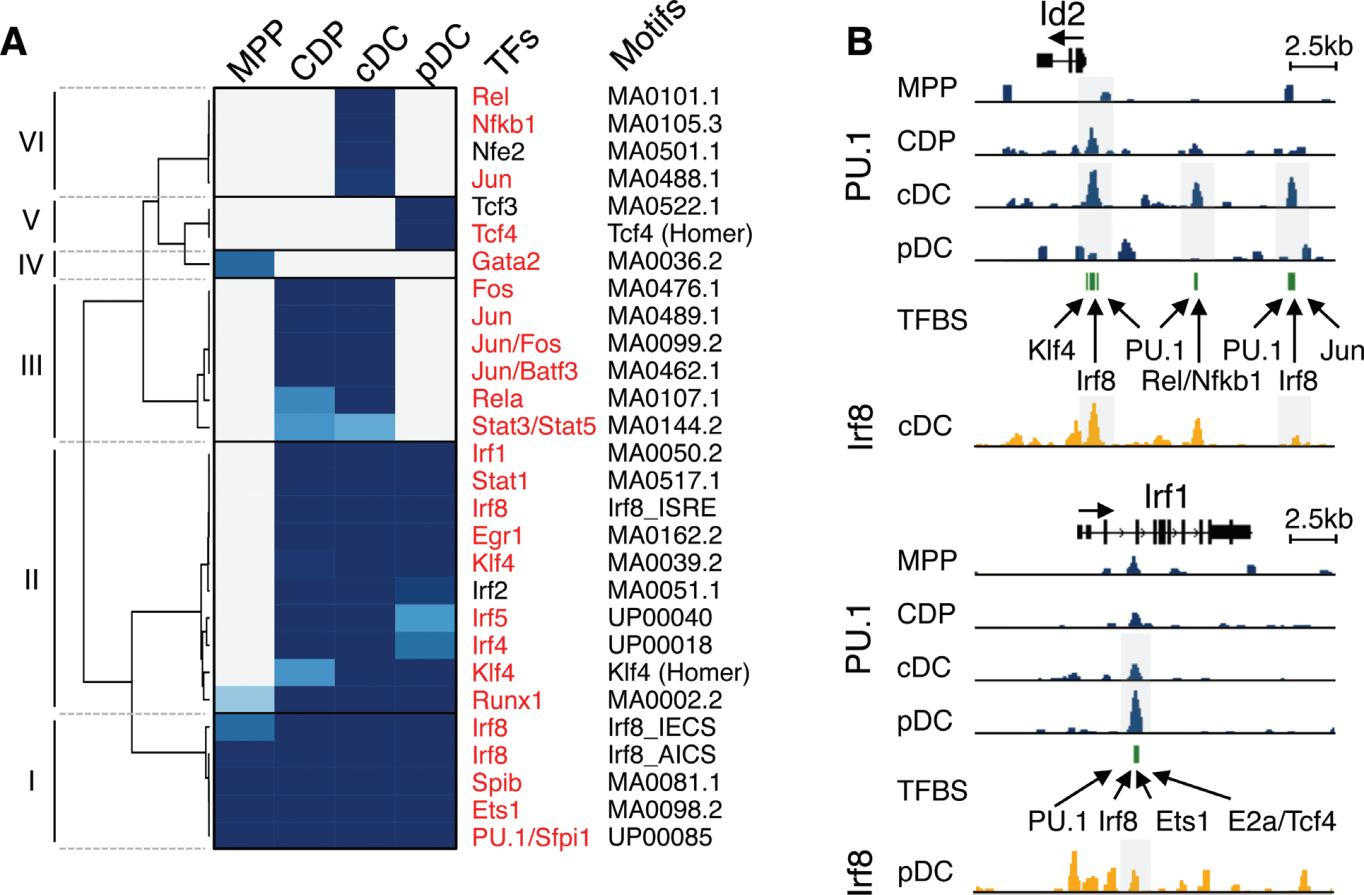
Identification of lineage-specific transcription factor regulatory networks. (**A**) Heat map depicts the enrichment of transcription factor motifs (row) in MPP, CDP, cDC and pDC (column). *P* values are plotted and color-coded using a continuous spectrum from gray (*p* value > 0.05) to blue (*p* value < 0.05). Twenty transcription factors that are considered as potential PU.1 co-binding partners are labeled in red. Clusters I to VI are indicated. (**B**) PU.1 and Irf8 occupancy of Id2 and Irf1 loci are shown for MPP, CDP, cDC and pDC. Differential PU.1 peaks and Irf8 peaks are highlighted by gray boxes. Green lines indicate transcription factor binding sites (TFBS) predicted inside differential PU.1 peak regions. Selected TFBS are labeled by gene symbol.

In total, 23 transcription factors (represented by 28 motifs) are significantly enriched at different stages of DC development (Figure 4A). Transcription factors with opposing expression and enrichment patterns (Supplementary Figure S6) were excluded from further analysis. Twenty transcription factors, including PU.1 itself (red in Figure 4A), were predicted as PU.1 partners and classified into 6 stage-specific clusters. Irf8 and Ets-domain transcription factor Spib (Cluster I; Figure 4A) are known interaction partners of PU.1 and also implicated in DC differentiation (3). Irf8-deficient mice lack many mature DC subsets. Spib is also indispensable for DC development, particular in pDC (57). Both factors show a significant enrichment in MPP, CDP and the two DC subtypes, indicating that they undergo composite binding with PU.1 in each step of DC development. Moreover, Gata2 is enriched only in MPP (Cluster IV), in line with cooperated functions of both Gata2 and PU.1 in myeloid cell fates (58).

Upon DC commitment a panel of transcription factors, such as Irf1/4/5/8, Stat1, Egr1, Klf4, and Runx1, are enriched in CDP (Cluster II; Figure 4A). This indicates that PU.1 initiates the DC program by recruiting or cooperating with multiple DC regulators, including known and novel PU.1 interacting factors. For example, up-regulation of Id2 expression is accompanied by an increase of PU.1 binding in the Id2 promoter from MPP to CDP (Figure 4B). Within this PU.1 binding region, PU.1, Irf8, Klf4 and Egr1 binding sites were detected, suggesting that these factors are recruited by PU.1 to promote Id2 expression.

Upon DC subset specification, PU.1 is predicted to collaborate with distinct sets of transcription factors to restrict the developmental program toward to either cDC or pDC, such as Rel/Nfkb1 for cDC and Tcf4 for pDC (Clusters III, V and VI; Figure 4A). The Id2 gene, a prototype cDC marker, contains specific PU.1 peaks at promoter and distal regions in cDC, which are associated with binding sites of PU.1, Jun, Rel/Nfkb, Irf8, Klf4 and Egr1 (Figure 4B). Conversely, the pDC-affiliated gene Irf1 contains PU.1 binding regions, which harbor the binding sites of Tcf4, a prototype pDC transcription factor, Irf8 and Ets1 (Figure 4B).

Frequently, PU.1 peaks harbor predicted Irf8 binding sites. Thus, to validate co-binding of PU.1 and Irf8, we generated Irf8 ChIP-seq data of cDC and pDC. We also included a recently published Irf8 ChIP-seq data set by Grajales-Reyes et al. (59). Irf8 ChIP-seq data validate the predicted Irf8 binding in Id2 and Irf1 genes in cDC and pDC (Figure 4B). Receiver operating characteristic (ROC) curves further show the accuracy of Irf8 binding site prediction within the PU.1 peaks (Supplementary Figure S7). Additionally, many cDC or pDC specific regulators identified in this analysis were found to be enriched in CDP (Figure 4A), such as Jun/Fos/Batf3 and Stat3/Stat5, again supporting the notion of DC priming in CDP.

Next we examined co-localization of PU.1 peaks and H3K4me1 footprints, which represent enhancer regions with potential transcription factor binding (46,60). We found an increasing overlap of PU.1 binding and H3K4me1 from 20% in MPP to 70% in cDC (Supplementary Figure S8A), indicating an increasing recruitment of PU.1 to enhancer elements during DC development. However, other enhancer elements did not show PU.1 occupancy. We therefore performed transcription factor motif enrichment analysis in H3K4me1 footprints with PU.1 peaks (Supplementary Figure S8B). The analysis captured many PU.1 co-binding transcription factors identified above (Figure 4A) and two additional factors (Cebpa and Bhlhe40), yet not the MPP factor Gata2 and the cDC-specific factors Jun/Batf3, Rel/Nfkb1 and Stat3/5. Further enrichment analysis in H3K4me1 footprints without PU.1 peaks identified much less transcription factors (Supplementary Figure S8C). Given the deficiency in capturing crucial cDC transcription factors (3) using only H3K4me1 footprints, we continued our analysis with the PU.1 co-binding factors identified in Figure 4A.

### Construction of cell-specific transcription factor regulatory networks

**Figure 5.**
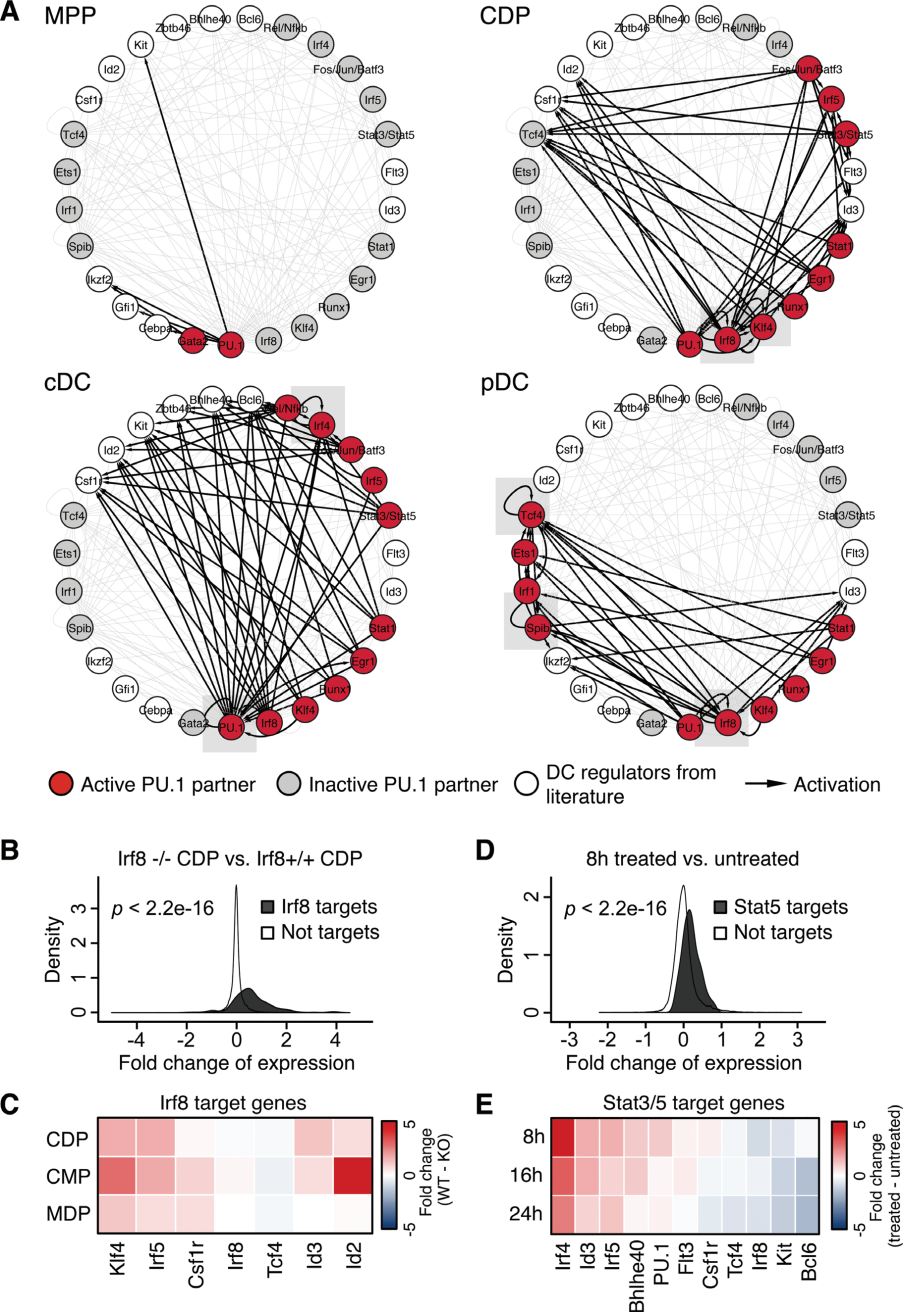
Construction and validation of stage-specific transcription factor regulatory networks. (**A**) Transcription factor regulatory networks of MPP, CDP, cDC and pDC. Nodes represent identified PU.1 co-binding partners from Figure 4A that are active (red) or inactive (gray) in MPP, CDP, cDC and pDC. Selected key DC regulators from the literature (white) were included in the analysis (6,39–45). A directed edge from transcription factor *a* to target gene *b* (black) indicates the association with gene activation, i.e., (i) the transcription factor is enriched in the respective cell type, (ii) the target gene is differentially expressed during DC commitment (MPP versus CDP) or DC subset specification (cDC versus pDC) and (iii) there is a transcription factor binding site at a differential PU.1 peak of the target gene. A self-loop edge (gray box) indicates an auto-regulatory feedback loop. The gray edges show regulatory links predicted in at least one of the networks. (**B**) The density plot of predicted Irf8 target genes shows higher gene expression in Irf8+/+ CDP than in Irf8−/− CDP. (**C**) Irf8 target genes in CDP of (A) are validated by comparing gene expression data of Irf8−/− and Irf8+/+ CDP, CMP and MDP (GSE34915, GSE45467). Red and blue, higher or lower gene expression, respectively, in Irf8+/+ than Irf8−/− cells. (**D**) The density plot of predicted Stat3/Stat5 target genes in Stat5-ER DC progenitors shows an increase of gene expression in response to 8h taxmoxifen (tmx) treatment. (**E**) Stat3/Stat5 target genes in CDP and cDC of (A) are validated using conditional Stat5-ER expression for 8, 16 and 24 hours. Red and blue, higher and lower gene expression in response to tamoxifen activated Stat5-ER, respectively. *P* values were calculated using Wilcoxon rank sum test.

We proceeded to construct cell-specific transcription factor regulatory networks (Figure 5A). In each network, nodes represent the potential PU.1 co-binding partners (Figure 4A) and selected key DC regulators (e.g. Flt3, c-Kit and Id2; (3,11)). An edge between two nodes indicates that a particular transcription factor is associated with activation of its target gene.

Different topologies and connectivity densities of the four stage-specific networks reflect how consecutive recruitment of PU.1 co-binding factors drives DC development (Figure 5A). In CDP, PU.1 recruits a core set of transcription factors (e.g. Irf8, Klf4, Runx1, Egr1 and Stat1) to activate the expression of DC marker genes (e.g. Id2, Csf1 and Tcf4; Figure 5A). In cDC and pDC, DC subtype-specific transcription factors collaborate with PU.1 to define cDC or pDC identity (Figure 5A). For example, Rel/Nfkb and Irf4 exclusively co-bind with PU.1 in cDC, whereas Tcf4, Ets1, Irf1 and Spib only cooperate with PU.1 in pDC.

Auto-regulatory feedback loops are important building blocks of transcriptional regulatory networks (61,62). PU.1 was shown to control hematopoietic development by forming auto-regulatory loops (63,64). Intriguingly, several positive auto-regulatory loops of key DC genes are captured in our networks (Figure 5A). The auto-regulatory loop of Irf8 in CDP indicates that Irf8 induces its own transcription, emphasizing the important function of Irf8 in DC commitment (Supplementary Figure S9). An auto-regulatory loop of Irf8 was also observed in pDC, which is in line with Irf8 being abundantly expressed in pDC and required for pDC development (65). Similarly, an auto-regulatory loop was also observed for Irf4 in cDC, which is in accordance with Irf4 function in cDC development (Supplementary Figure S9; (3)).

To confirm predicted network connections, we performed functional tests for Irf8 and Stat5 (Figure 5B–E). The predicted Irf8 target genes show higher gene expression in Irf8+/+ CDP compared to Irf8−/− CDP (Figure 5B and C), which is further supported by Irf8 knockout studies in common myeloid progenitors (CMP) and macrophage dendritic cell progenitors (MDP) (Figure 5C). Similarly, Stat3/Stat5 targets in Stat5-ER DC progenitors show an overall up-regulation in response to tamoxifen treatment compared to untreated cells (Figure 5D and E).

### Transcription factor regulatory circuitry of DC development

**Figure 6.**
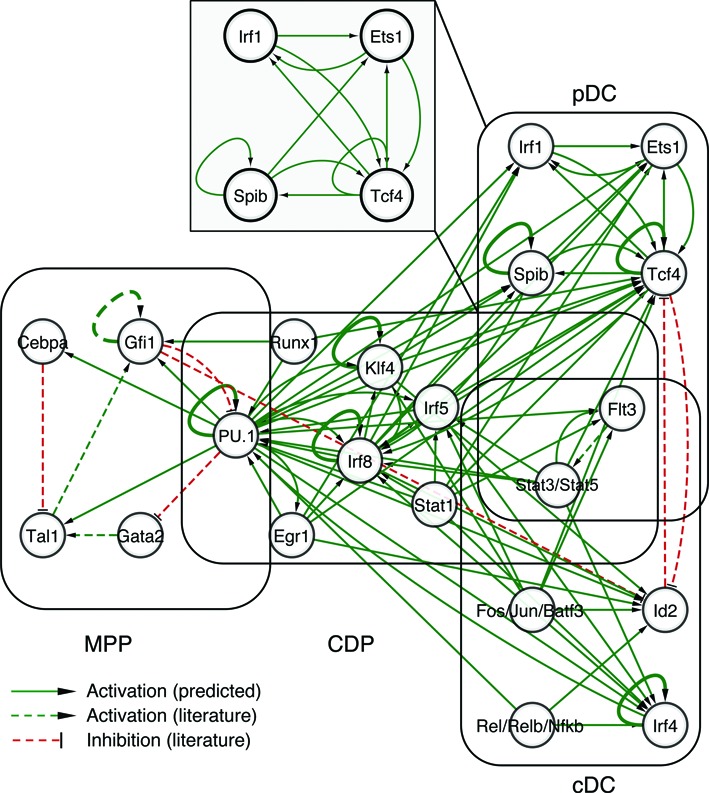
The regulatory circuitry of DC development. The network illustration depicts the organization of the integrated DC regulatory circuitry. Nodes represent key regulatory factors involved in DC development. A directed edge from factor *a* to factor *b* indicates an active function (green) or inhibition function (red) of factor *a* to factor *b*. Dotted edges represent additional interactions obtained from the literature (6,39–45). The pDC sub-network of Irf1, Ets1, Spib and Tcf4 is shown (gray box).

We proceeded to integrate the four stage-specific transcription factor networks in one DC regulatory circuitry (Figure 6). Each regulator in the circuitry is positioned at a specific stage of DC development in which it is involved. Additionally, some of the positive or negative interactions between DC regulators were included based on the literature (6,39–45). For example, PU.1 was observed to inhibit Gata1/2 activation, which led to specific myeloid cell fates (58). All key regulators of the circuitry play crucial roles in DC development, which is in line with gene knockout studies (Supplementary Figure S9A; (1)). Furthermore, the connectivity of a factor also relates with its function in DC development (Supplementary Figure S9B–H).

During MPP–CDP transition, PU.1 is connected with Flt3, Stat1/3/5, Irf5/8, Klf4 and Egr1, suggesting that it induces in concert with these factors a genetic program to initiate and establish DC lineage fate (Figure 6; Supplementary Figure S9B). Additionally, PU.1 appears to restrict MPP-CDP transition by directly or indirectly inhibiting alternative lineage fates, e.g. by inhibiting Gata2 (58). Following DC commitment, CDP can undergo two different developmental options: the subset specification into cDC and pDC. DC subset-specific factors control the antagonized developmental pathways leading to either cDC or pDC. For example, high expression of the cDC marker Id2 inhibits the pDC gene Tcf4 and vice versa, resulting in cDC or pDC development, respectively (10,66).

**Figure 7.**
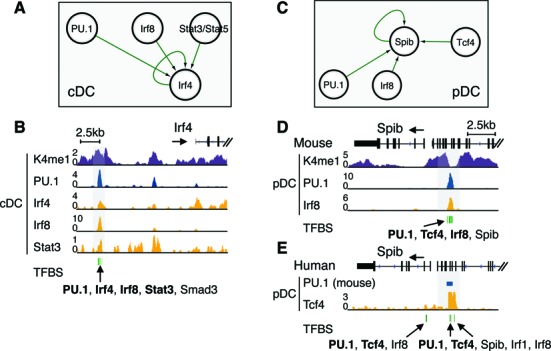
Sub-network of Irf4 and Spib by ChIP-seq analysis. (**A**) PU.1, Irf8 and Stat3/Stat5 are predicted to impact on Irf4 in cDC. The auto-regulatory loop of Irf4 is indicated (see network in Figure 6). (**B**) Occupancy of PU.1, Irf4, Irf8 and Stat3 in the enhancer region (H3K4me1) of Irf4 gene in cDC (highlighted in gray box) verify the predicted transcription factor binding sites (green bars). The ChIP-seq data of Irf4, Irf8 and Stat3 in cDC are from GSE36104, GSE53311 and GSE27161. (**C**) PU.1, Irf8 and Tcf4 are predicted to impact on Spib in pDC. The auto-regulatory loop of Spib is also indicated. (**D**) Occupancy of PU.1, Irf8 in Spib enhancer region (H3K4me1) in pDC (highlighted in gray box) is in line with the predicted co-binding of Irf8 and PU.1 (green bars). (**E**) The PU.1 peak in mouse pDC is mapped to human genome using UCSC liftOver tool and shown as blue bar. The PU.1 peak collocates with Tcf4 in human pDC (highlighted in gray box) and predicted transcription factor binding sites for PU.1, Tcf4, Spib, Irf1 and Irf8 (green bars). The ChIP-seq data of Irf8 and Tcf4 in pDC are from GSE62702 and GSE43876.

In the DC circuitry, the cDC marker Irf4 is predicted to be regulated by multiple transcription factors, including PU.1, Irf8, Stat3 and Irf4 itself (Figures 6 and 7). ChIP-seq data demonstrate PU.1, Irf4, Irf8 and Stat3 co-binding in the enhancer region of the Irf4 gene (Figure 7A and B), which provides experimental evidence for the predicted interactions within the network. Furthermore, Irf4 binding in the promoter and enhancer regions of Irf4 gene supports the auto-regulatory loop inferred for Irf4 (Figure 7A and B).

Multiple feedback loops were observed between pDC factors, such as Irf1, Ets1, Spib and Tcf4 (pDC sub-network in Figure 6), yet such feedback loops were not shown for cDC factors (Fos/Jun/Batf3, Rel/Relb/Nfkb, Id2 and Irf4). ChIP-seq data demonstrate PU.1, Irf8 and Tcf4 co-binding in the enhancer regions of Spib and Irf1, two key pDC genes (Figure 7C–E; Supplementary Figure S10). Collectively, our study reveals multiple interactions among the Irf1, Ets1, Spib and Tcf4 transcription factors, which might be indicative for reinforcement of the pDC program and underpins the hypothesis that cDC is the default DC development pathway. Branching off the default pathway and establishing the pDC subset require specific regulatory mechanisms to stabilize the pDC program.

## DISCUSSION

Here, we generated high-resolution genome-wide maps of H3K4me1, H3K4me3, H3K27me3 and PU.1 for DC commitment and subset specification. We demonstrate that consecutive changes of stage-specific expression for key DC regulators, including PU.1, Irf1, Irf8, Batf3, Spib and Tcf4, are associated with specific histone modifications in promoter and enhancer sequences. Genome-wide analysis led us to devise a regulatory circuitry, which provides the basic architecture of how DC transcription factors are wired to drive DC development.

Genes that are increasingly expressed during DC development show an increase in H3K4me1 and H3K4me3 marks and a decrease in H3K27me3, as expected. Interestingly, gain in H3K4me1 or H3K4me3 marks on DC genes activates a DC-primed gene expression profile in CDP, which results in DC lineage commitment. These results are in line with our data on the DC-primed transcriptional signature in CDP (5). Additionally, changes of bivalent domains lead to activation or repression of the DC-primed gene signature in CDP and of DC subset-specific genes in DC, suggesting an important role of bivalent marks in DC commitment and specification.

PU.1 represents a pioneer transcription factor in hematopoietic cell development that acts in concert with other lineage specific factors (25,26,49,67). DC progenitors and DC subsets express PU.1 and knockout mice demonstrated the impact of PU.1 on DC development (68,69). PU.1 controls Flt3 cytokine receptor expression and Flt3/Stat signaling induces PU.1 expression, thus generating a self-reinforcing auto regulatory loop that drives DC development (6,47). PU.1 also induces chromatin remodeling of the Irf8 gene that encodes an important transcription factor for DC development (14). Our genome-wide analysis of PU.1 occupancy is very much in line with the crucial role of PU.1 in DC development. First, we identified an alternative PU.1 motif in MPP compared to CDP, cDC and pDC. Second, PU.1 is predicted to associate with stage-specific transcription factors during the sequel MPP-CDP-cDC/pDC. Both PU.1 binding to stage-specific cis-regulatory elements and recruitment of stage-specific co-binding transcription factors translate into activation of specific target genes. Moreover, the predicted PU.1 co-binding transcription factors include key DC regulators, such as Irf family members (i.e. Irf1, Irf4, Irf5 and Irf8), Klf4, Spib and Tcf4. For example, the co-binding of Tcf4 and PU.1 was specifically observed in pDC, while Irf4 and PU.1 co-binding was confined to cDC. Additionally, PU.1 can also repress gene expression, such as Gata2 expression, and thereby affect cell differentiation (58). Thus, both positive and negative activities of PU.1 are important for directing cell fate.

Previous work proposed a layered transcription factor network for inflammatory DC stimulated with lipopolysaccharide (26). PU.1 was also positioned at the top of this transcription factor network. However, compared to Garber *et al*. (26), our regulatory circuitry reveals the transcription factor architecture for the entire sequel MPP–CDP–cDC/pDC, thus covering all consecutive stages of DC development. Importantly, the positions and inter-connections of transcription factors within the circuitry reveal their activity in both DC lineage commitment and subsets specification. Additionally, our regulatory circuitry describes the successive stages of DC development in the steady state, while previous work studied inflammatory DC.

Here, we addressed an emerging need for network construction from gene expression and ChIP-seq data (22) and developed an integrative approach by combining differential transcription factor binding, gene expression data and motif enrichment analysis. This allowed us to reverse engineer a transcriptional network model that captures multiple feedback loops of key DC regulators. This transcription factor circuitry for DC development is very much in accord with the large array of gene knockout studies (Supplementary Figure S9A; (1)). Along those lines, we validated targets inferred from transcription factor binding predictions. For example, we demonstrate that predicted Irf8 target genes are more abundantly expressed in Irf8+/+ cells than Irf8−/− cells. However, such functional studies appear to be more complex than anticipated. This is because in regulatory networks nodes receive input from several directions and thus manipulations by only one factor might cause only limited effects. Additionally, the impact of perturbations needs to be measured shortly after transcription factor activation (or inhibition) to avoid secondary and indirect regulation of targets. We addressed this issue by employing a conditional StatER construct, which allows a time-dependent target gene activation. We show induction of predicted Stat3/Stat5 target genes; however, some Stat3/Stat5 target genes were not or only marginally affected, suggesting that further factors are involved.

Importantly, several auto-regulatory feedback loops were identified for key DC genes, such as Irf4, Irf8, Klf4, Tcf4 and Spib. ChIP-seq data demonstrate co-localization of PU.1, Irf8, Stat3 and Irf4 at upstream region of Irf4 gene. This supports the predicted gene-target connections in DC transcription factor circuitry, including the auto-regulatory loop of Irf4 inferred from the prediction. Such auto-regulatory loops reinforce the expression of stage-specific transcription factors and lock cells in specific differentiation stages, thereby leading to the overall stabilization of the network. Additionally, in keeping with cDC being considered as the default DC developmental pathway (3), we suggest a specific pDC subnetwork (Tcf4, Spib, Irf1 and Ets1) containing multiple feedback loops. This pDC circuitry is predicted to allow pDC to branch off from the cDC default pathway and to stabilize pDC identity.

In summary, here we describe the basic architecture of the DC transcriptional regulatory program, which drives commitment of hematopoietic stem cells and their differentiation into DC. We provide a comprehensive characterization of the interplay between individual key DC regulators at specific stages of DC development, which is expected to pave the way for specific tailoring of DC development and function.

## Supplementary Material

SUPPLEMENTARY DATA
