# Supplementary material for: Epigenetic program and transcription factor circuitry of dendritic cell development
Source: Nucleic Acids Res. 2015 Oct 17;43(20):9680–93. doi: 10.1093/nar/gkv1056 (PMC4787753; doi:10.1093/nar/gkv1056)
Supplement: SUPPLEMENTARY DATA [file supp_43_20_9680__index.html]

Epigenetic program and transcription factor circuitry of dendritic cell development — Epigenetic program and transcription factor circuitry of dendritic cell development — Epigenetic program and transcription factor circuitry of dendritic cell development — SUPPLEMENTARY DATA 

# Epigenetic program and transcription factor circuitry of dendritic cell development

## SUPPLEMENTARY DATA

- SUPPLEMENTARY DATA
- SUPPLEMENTARY DATA
