# Supplementary material for: Epigenetic program and transcription factor circuitry of dendritic cell development
Source: Nucleic Acids Res. 2015 Oct 17;43(20):9680–93. doi: 10.1093/nar/gkv1056 (PMC4787753; doi:10.1093/nar/gkv1056)
Supplement: SUPPLEMENTARY DATA [file supp_gkv1056_Suppl_data_gkv1056.pdf]

Supplementary Data

Epigenetic Program and Transcription Factor Circuitry of Dendritic Cell Development

Qiong Lin[1,2,†], Heike Chauvistré[1,2,3,†], Ivan G. Costa[4,5,†], Eduardo G. Gusmao[4], Saskia Mitzka[1,2], Sonja Hänzelmann[4], Bianka Baying[6], Theresa Klisch[1,2], Richard Moriggl[7], Benoit Hennuy[8], Hubert Smeets[9,10], Kurt Hoffmann[11], Vladimir Benes[6], Kristin Seré[1,2] and Martin Zenke[1,2,*]

[1] Institute for Biomedical Engineering, Department of Cell Biology, RWTH Aachen University Medical School, 52074 Aachen, Germany
[2] Helmholtz Institute for Biomedical Engineering, RWTH Aachen University, 52074 Aachen, Germany
[3] Department of Dermatology, University Hospital Essen, 45147 Essen, Germany
[4] IZKF Computational Biology Research Group, RWTH Aachen University Medical School, 52074 Aachen, Germany
[5] Aachen Institute for Advanced Study in Computational Engineering Science (AICES), RWTH Aachen University, 52062 Aachen, Germany
[6] Genomics Core Facilities GeneCore, European Molecular Biology Laboratory (EMBL), 69117 Heidelberg, Germany
[7] Ludwig Boltzmann Institute for Cancer Research, University of Veterinary Medicine, Medical University Vienna, 1090 Vienna, Austria
[8] GIGA-Genomics, University of Liège, 4000 Liège, Belgium
[9] Department of Genetics and Cell Biology, Maastricht University Medical Center, 6200 MD Maastricht, The Netherlands
[10] Research Schools CARUM and GROW, Maastricht University Medical Center, 6200 MD Maastricht, The Netherlands
[11] Institute of Molecular Biotechnology, RWTH Aachen University, 52074 Aachen, Germany
* To whom correspondence should be addressed. Tel: +49-241-8080760; Fax: +49-241-8082008; Email: martin.zenke@rwth-aachen.de. † These authors contributed equally to this work.

Supplementary Methods **2**
ChIP-seq assay 2
The differential PU.1 binding analysis 2
Identification of PU.1 co-binding transcription factors 3
Construction of DC regulatory networks 4
Detection of H3K4me1 footprints 5

Supplementary Figures **6**
Supplementary Figure S1. Dynamics of gene expression during DC development *in vivo* in FACS sorted progenitors and DC subsets. 6
Supplementary Figure S2. Epigenetic regulation during DC commitment. 7
Supplementary Figure S3. Epigenetic regulation during DC subset specification. 8
Supplementary Figure S4. PU.1 peaks in promoters and enhancers. 9
Supplementary Figure S5. Workflow of regulatory network inference analysis. 10
Supplementary Figure S6. Gene expression of PU.1 co-binding transcription factors. 11
Supplementary Figure S7. ROC analysis of predicted Irf8 binding sites. 12
Supplementary Figure S8. Enrichment of transcription factor motifs for enhancer elements. 13
Supplementary Figure S9. Knockout phenotype of key genes in the regulatory circuitry of DC development. 14
Supplementary Figure S10. Cobinding of PU.1, Irf8 and Tcf4 in Irf1 gene in pDC. 15

Supplementary References **16**

Supplementary Methods

ChIP-seq assay

Sorted cells were cross-linked at a concentration of 2 million cells/ml with 1% formaldehyde for 6 min at room temperature. Cross-linking was stopped with 0.125 M glycine. Chromatin sonification into 200-400 bp fragment size was done with Bioruptor with cooling device (Diagenode) at 4°C for 10 min with 30s pulse/pause cycles. Sheared lysates were clarified by centrifugation (12,000g, 10 min, 4°C). 10 µl Dynabeads Protein A (Life Technologies) were preincubated with either 1 µg IgG control (Santa Cruz Biotechnology) or specific antibodies for H3K4me1 (Abcam, ab8895), H3K4me3 (Diagenode, pAb-003-050), H3K27me3 (Diagenode, pAb-069-050) and PU.1 (Santa Cruz, sc-352).

For immunoprecipitation, sheared chromatin of 1 million cells was added to the preincubated beads over night at 4°C. Chromatin complexes were isolated by magnetic bead selection and washed with RIPA and TE buffer. Chromatin complexes were digested with 50 µg/ml RNase (Roche) at 37°C for 30 min. Immunoprecipitated DNA was purified using QIAquick PCR Purification Kit according to the manufacturer's protocol (Qiagen). DNA concentration of immunoprecipitated DNA was determined by using Qubit dsDNA HS Assay kit (Life Technologies). Libraries were prepared and subjected to deep-sequencing on the Illumina platform according to the manufacturer's protocols.

The differential PU.1 binding analysis

To detect differential PU.1 binding, the PU.1 peaks in MPP, CDP, cDC and pDC were merged to generate a complete set of peaks. The coverage of reads within the peaks in the complete set was used to estimate the peak signal. The differential PU.1 peaks between pairs of cell types (MPP versus CDP and cDC versus pDC) were detected using exact

empirical test from EdgeR (1). Peaks with low read support (i.e., counts-per-million < 3.0) were excluded from further analysis. A peak was considered differential if it produced a significant fold change and *p* value < 0.01 after Benjamini-Hochberg multiple test correction (2). *De novo* motif detection for PU.1 was performed with MEME-ChIP (3) by providing 200bp regions around the summits of differential peaks. The motif with highest number of binding sites was reported.

Identification of PU.1 co-binding transcription factors

First, differentially expressed genes upon DC commitment (MPP versus CDP) and specification (cDC versus pDC) were detected described as above (Supplementary Figure S5A). Second, transcription factor motifs were collected from Jaspar (4), Uniprobe (5) and Homer (6) (Supplementary Figure S5B). AICS Irf8 motif was obtained by applying the tool MEME-ChIP with default parameters (3) to Irf8 ChIP-seq peaks (7). The Irf8 ISRE and IEACS motifs were obtained from the sequences provided in the literature (8). The motifs of transcription factors with low gene expression or low variation upon DC development were excluded from further analysis.

Next, peaks were assigned to genes if they were in the proximal promoter (1kb upstream of the TSS) and in the gene body (Supplementary Figure 5C). To detect distal peaks, we also associated peaks when they were 50kb around the TSS and there was no other gene in between the TSS and the peak. Binding site detection was then performed within PU.1 differential peaks close to differentially expressed genes on the same cell type. All differential peaks were corrected to have uniform size, i.e., 250bp +/- the peak summit. Motif search was based on Biopython (9), utilizing the distribution of the information content of each motif to define a bit score threshold on the basis of a false discovery rate (FDR) test (Supplementary Figure S5D). We used the FDR value of 0.1 for all binding sites.

We selected random genomic regions with replacement from the mouse genome (mm9) and devised a method to make random regions to have the same proportion of CG content and mappability characteristic as DNA regions inside PU.1 peaks. For that, the whole genome was split into bins of 1000 bps. Then, the proportions of CpGs (ratio between the number of CpGs and the sum of the number of Cs and Gs) and unmappable regions (number of base pairs that overlap with regions that occur four or more times in the genome) were evaluated for each bin. Finally, the filtered random data set is built by randomly selecting bins with up to 1% difference of CpG and unmappable regions proportion from the regions of differential PU.1 binding. The unmappable regions were obtained upon processing the alignability data set (50bp window) from the mappability track of the ENCODE repository and blacklisted genomic regions (10). The final number of regions equals 10 times the number of regions in the largest differential PU.1 peaks data set.

Finally, we employed a one-tailed Fisher's exact test to measure if the proportion of differential PU.1 peaks close to differentially expressed genes with at least one transcription factor binding site is higher than the proportion of binding sites in random regions. The test was repeated for all motifs and cell-specific differential peaks. Final *p* values were corrected using the Benjamini-Hochberg method (2). The corrected *p* values (or enrichment scores) were visualized in heat map format (Figure 4A; Supplementary Figure S5E). The transcription factors with p value < 0.05 were predicted as PU.1 co-binding partners.

Construction of DC regulatory networks

The PU.1 co-binding transcription factors identified above and the key DC regulators selected from the literature (11-18) were used to build lineage-specific transcription factor networks (Figure 5; Supplementary Figure S5F). For MPP and CDP networks, differentially regulated genes during DC commitment (MPP versus CDP) were considered. For cDC and pDC networks, differentially regulated genes during DC subset specification (cDC versus

pDC) were considered. In each network, nodes represent transcription factors or genes that are connected by edges if the transcription factor (source) is predicted to bind to the gene (source) in the previous analysis. Binding sites of transcription factors enriched in the particular cellular context are depicted in black. In addition, nodes are color-coded differently, i.e., enriched transcription factors in red; non-enriched transcription factors in gray; selected key genes in white. Networks were generated by Cytoscape (19).

Detection of H3K4me1 footprints

Transcription factor binding sites are likely to occur between two regions with high levels of active histone marks (20,21), referred to as footprints. We employed a modified version of our computational method described in Gusmao et al. (21) to detect significant footprints in H3K4me1 ChIP-seq data. Briefly, the number of states of the hidden Markov model was reduced to include only background, histone level and footprint states. Additional transitions were added from the histone level DOWN state to the FOOTPRINT state and from the FOOTPRINT state to the histone-level UP state. The parameters of the model were estimated using H3K4me1 ChIP-seq data from random genomic regions. Given the lower resolution of ChIP-seq data and the nature of the probabilistic model, footprints from H3K4me1 tend to span larger regions. Therefore, we further reduced the footprint by considering only 250 bp upstream and downstream of its center.

Supplementary Figures

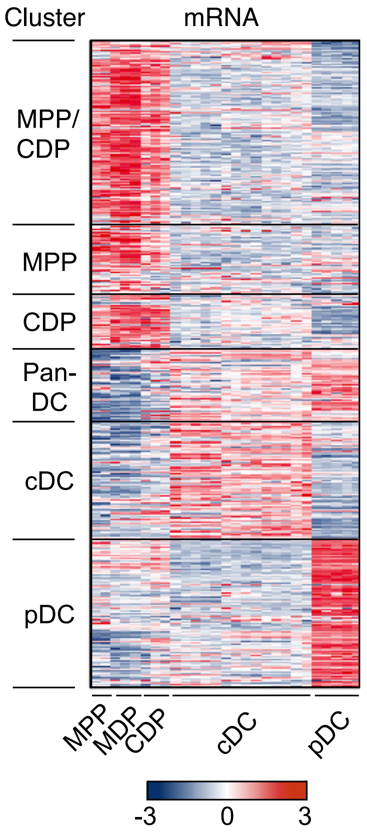

Supplementary Figure S1. Dynamics of gene expression during DC development *in vivo* in FACS sorted progenitors and DC subsets.

Heat map representation of gene expression (mRNA) in MPP, MDP, CDP, cDC and pDC obtained by FACS sorting from mice using the same 3194 differentially expressed genes as in Figure 1A. MPP, MDP and CDP, 2, 3 and 3 replicates, respectively; cDC, a panel of $CD8^+$, $CD8^-CD4^+$, $CD8^-CD4^-CD11b^+$ subsets in 5, 4 and 5 replicates, respectively; pDC in 5 replicates (GSE15907; (22)) .Red, high expression; blue, low expression.

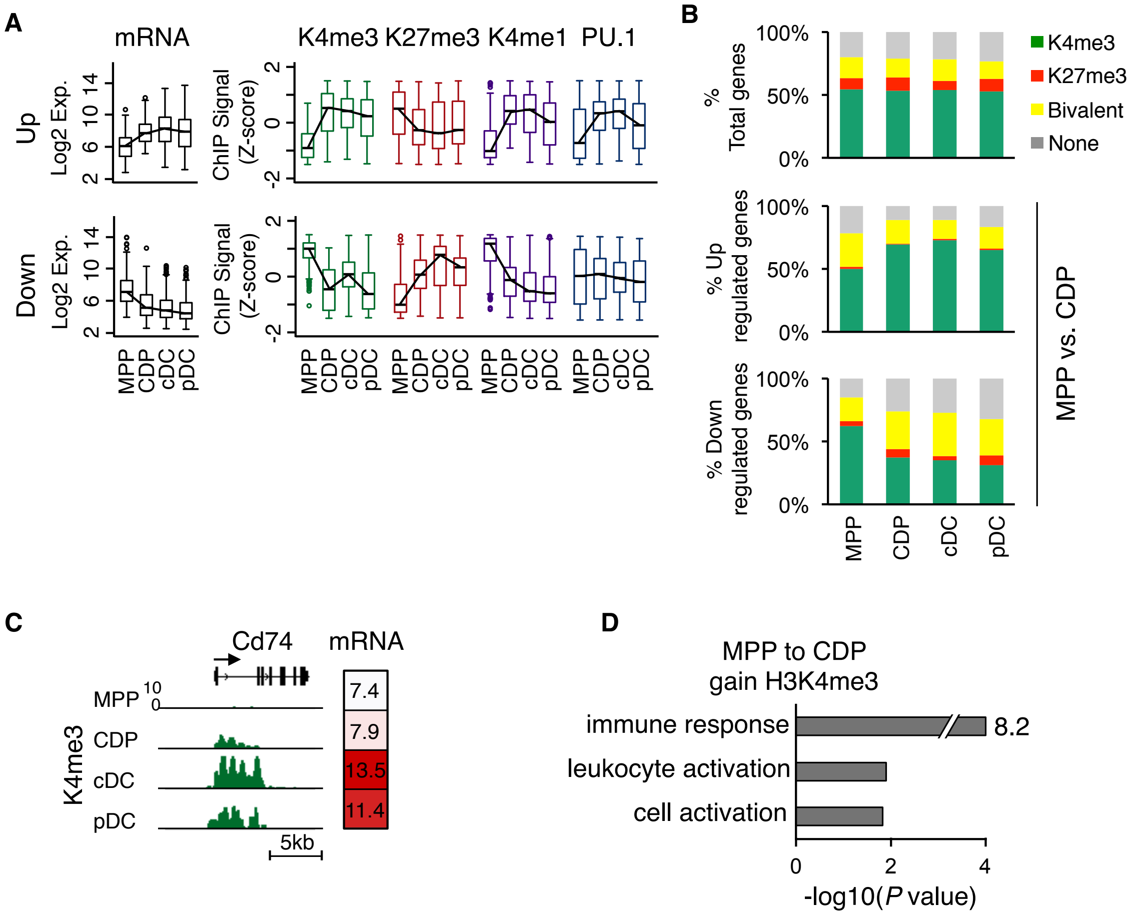

Supplementary Figure S2. Epigenetic regulation during DC commitment.
(A) Boxplot analysis of mRNA expression and of H3K4me3, H3K27me3, H3K4me1 and PU.1 occupancy of up or down-regulated genes from MPP to CDP (218 and 211 genes, respectively). Changes across DC development (MPP, CDP, cDC and pDC) are shown. H3K4me3 and H3K27me3, TSS±1kb; H3K4me1 and PU.1, TSS±50kb. **(B)** The percentage of genes with H3K4me3, H3K27me3 or both (bivalent domain) or no modifications is shown. All genes, top panel; Up and down-regulated genes between MPP and CDP, middle and low panel, respectively. **(C)** H3K4me3 occupancy and mRNA profiles of Cd74 gene (MHC class II invariant chain), which is up-regulated upon DC development. **(D)** Enriched gene ontology terms of genes, which gain H3K4me3 from MPP to CDP.

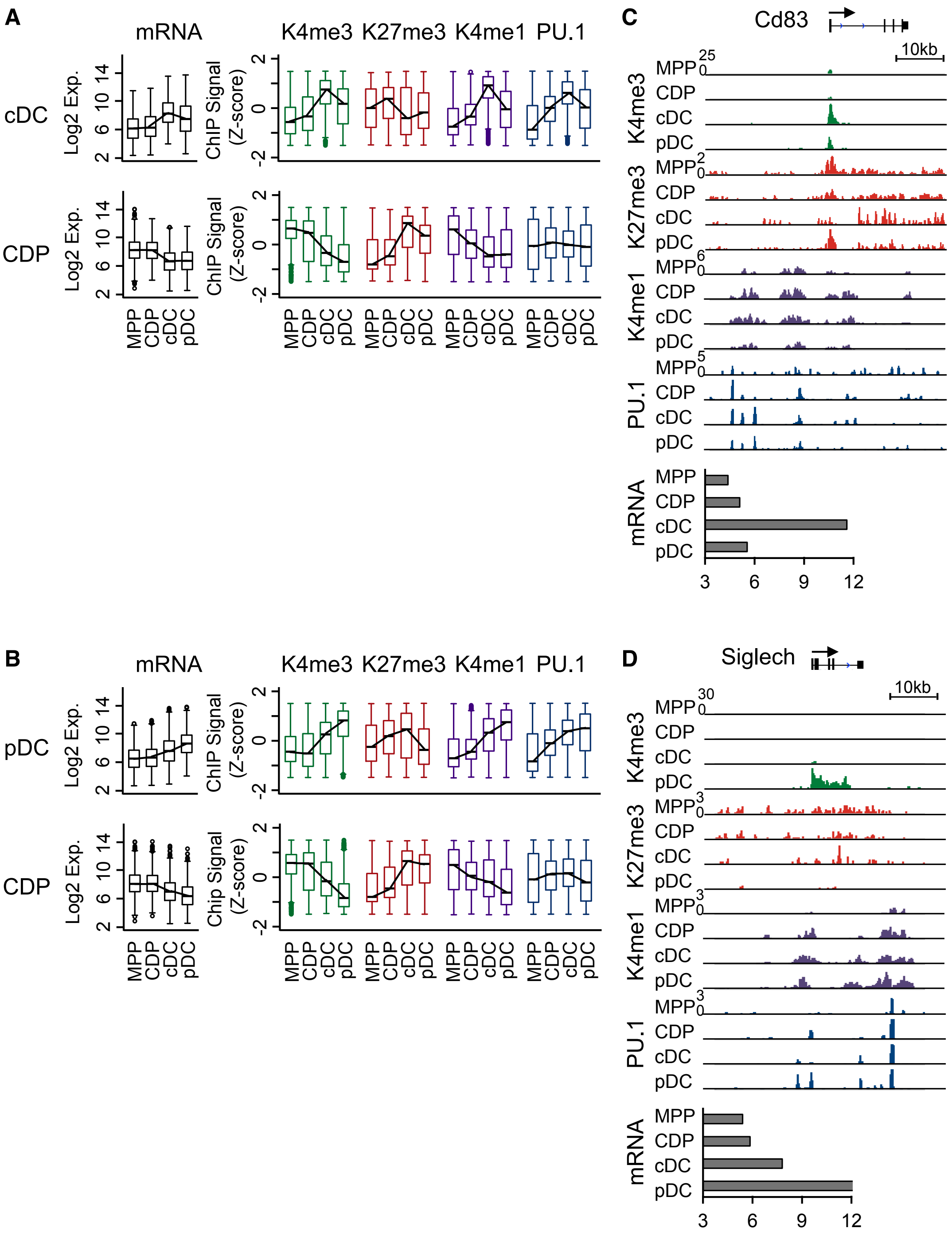

Supplementary Figure S3. Epigenetic regulation during DC subset specification.
(A-B) Boxplot analysis of mRNA expression and of H3K4me1, H3K4me3, H3K27me3 and PU.1 occupancy of differentially expressed genes during CDP-cDC transition (1773 genes, Figure 1A) and CDP-pDC transition (2181 genes, Figure 1A). Changes across DC development (MPP, CDP, cDC and pDC) are shown. H3K4me3 and H3K27me3, TSS±1kb; H3K4me1 and PU.1, TSS±50kb. **(C-D)** Occupancy for H3K4me1, H3K4me3, H3K27me3 and PU.1 and mRNA expression profiles (log2 expression) of the cDC gene Cd83 and the pDC gene Siglech in MPP, CDP, cDC and pDC. Arrow indicates the direction of transcription.

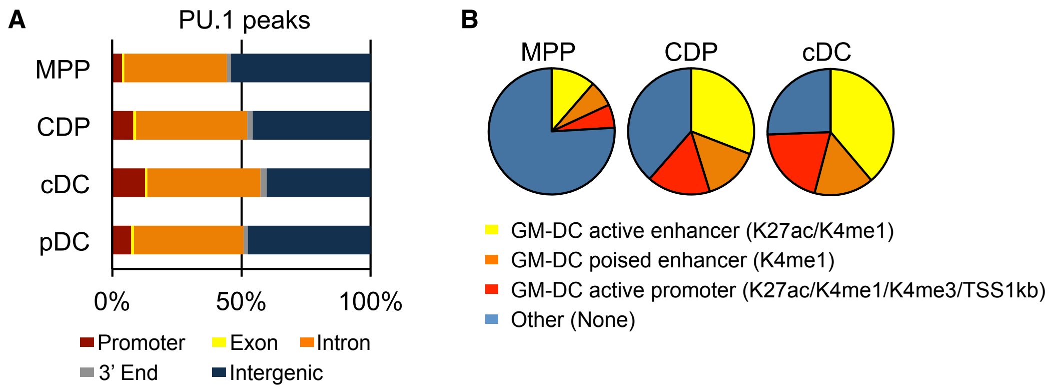

Supplementary Figure S4. PU.1 peaks in promoters and enhancers.
(A) The genomic distribution of PU.1 peaks in DC progenitors (MPP, CDP) and subsets (cDC, pDC). Promoter, TSS±1kb; 3' Ends, TES±1kb. **(B)** PU.1 peaks in active enhancers (regions modified by both H3K4me1 and H3K27ac), poised enhancers (regions only modified by H3K4me1) and active promoters (regions modified by H3K4me1, H3K4me3, H3K27ac and close to TSS±1kb) in MPP, CDP and cDC are shown. Others refers to PU.1 peaks without these histone modifications. The information on enhancer and promoter annotation was obtained from ChIP-seq analysis of GM-DC (GSE36104; (23)).

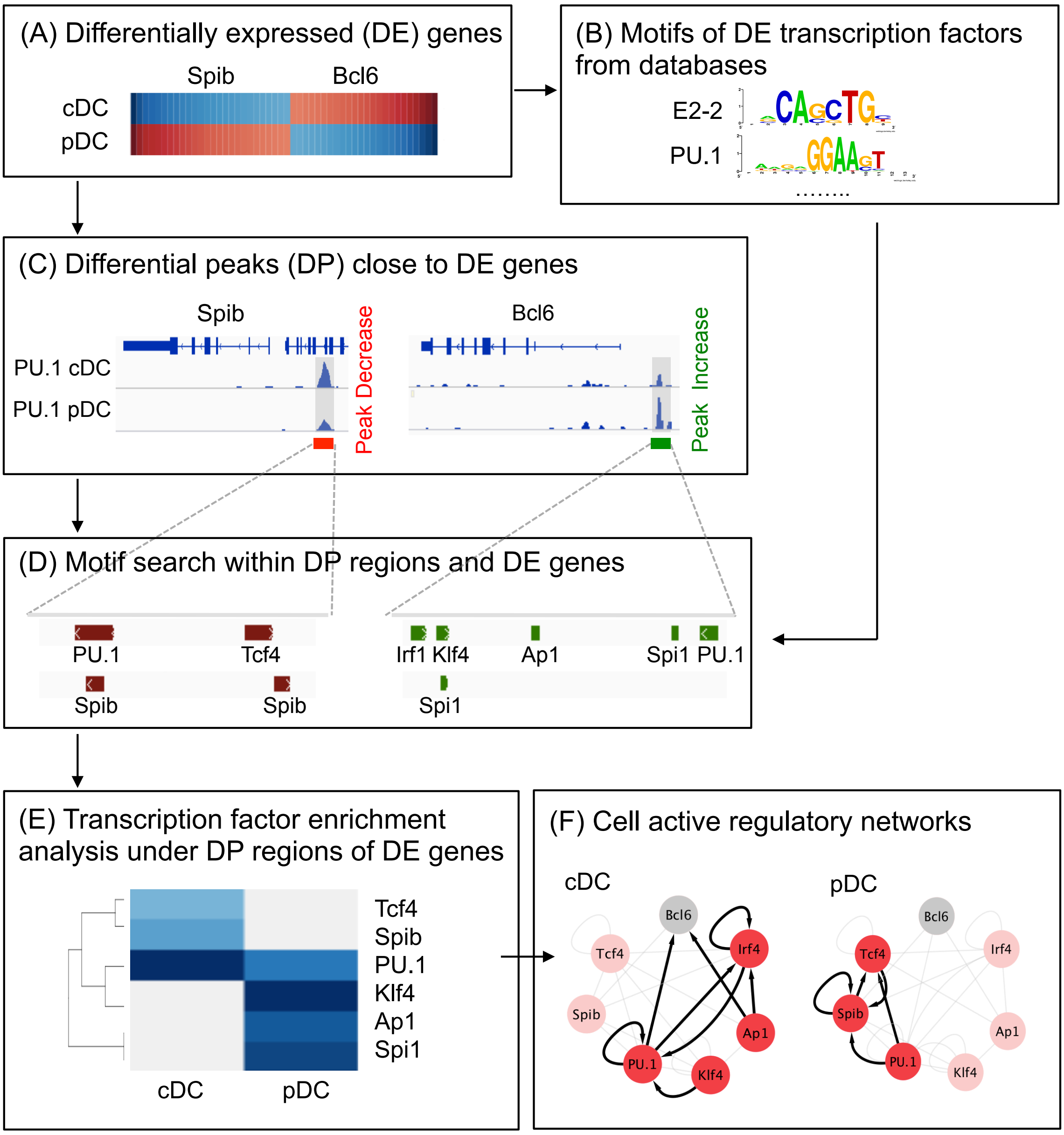

Supplementary Figure S5. Workflow of regulatory network inference analysis. **(A)** First we compiled all potential transcription factors and genes from the list of differentially expressed (DE) genes between two cell types, i.e. cDC versus pDC. **(B)** Next, we collected motifs from differentially expressed (DE) transcription factors (TFs) from public databases. **(C)** We identify differential peaks (DP; i.e. PU.1 peaks) close to differentially expressed (DE) genes of (A) in the same cell type, i.e. cDC differential peaks close to cDC differential genes. **(D)** Motif search from (B) within identified peak regions of (C). **(E)** Enrichment analysis of transcription factor motifs within differential peaks (DP) of (C) indicates transcription factors co-binding with PU.1 in a cell type-specific manner. **(F)** Construction of cell type-specific transcription factor regulatory networks by connecting enriched transcription factors to its putative targets. For example, Ap1 has an edge to Bcl6 because: Bcl6 is up-regulated in cDC, Bcl6 has a Ap1 binding site on a cDC differential peak close to its promoter and Ap1 binding is enriched within cDC differential peaks.

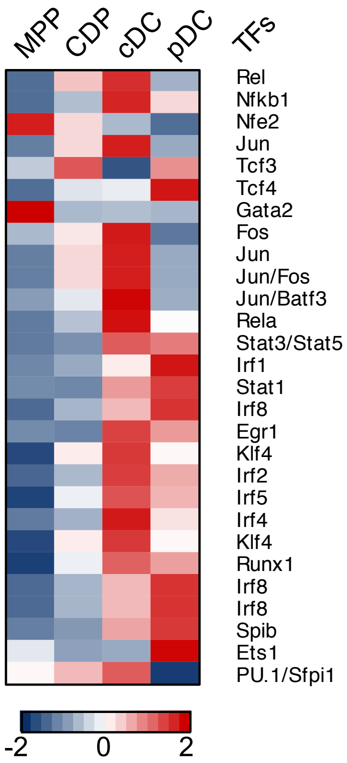

Supplementary Figure S6. Gene expression of PU.1 co-binding transcription factors. Gene expression of potential PU.1 co-binding transcription factors of Figure 4A in MPP, CDP, cDC and pDC is depicted in heatmap format. Red, high expression; blue, low expression.

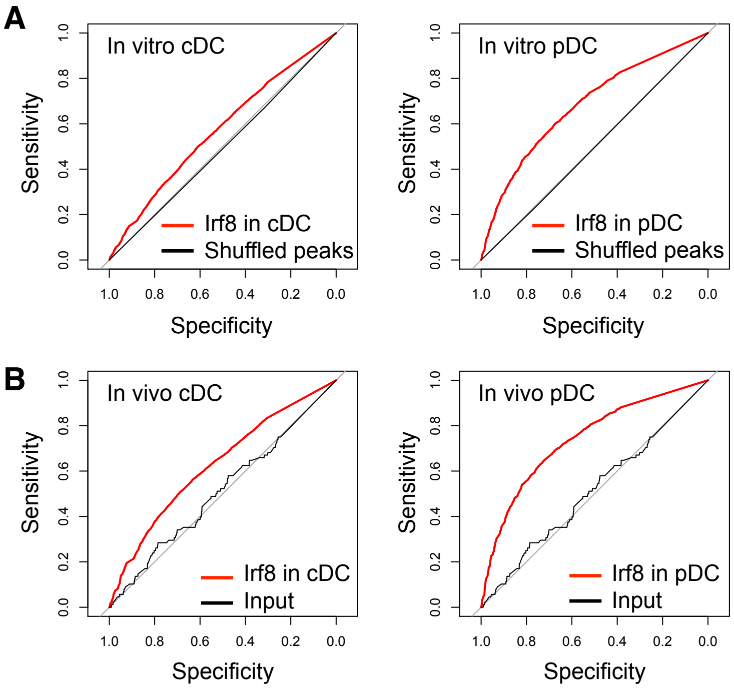

Supplementary Figure S7. ROC analysis of predicted Irf8 binding sites.
(A) ROC curve analysis of predicted Irf8 binding sites within PU.1 peaks in cDC or pDC using Ir8 ChIP-seq data from *in vitro* cDC and pDC (red). The random shuffled Irf8 peaks served as background control (black). **(B)** ROC curve analysis of predicted Irf8 binding sites within PU.1 peaks in cDC or pDC using Ir8 ChIP-seq data from *in vivo* cDC and pDC (red; GSE66899). Peaks identified in the input ChIP-seq data were used as control (black).

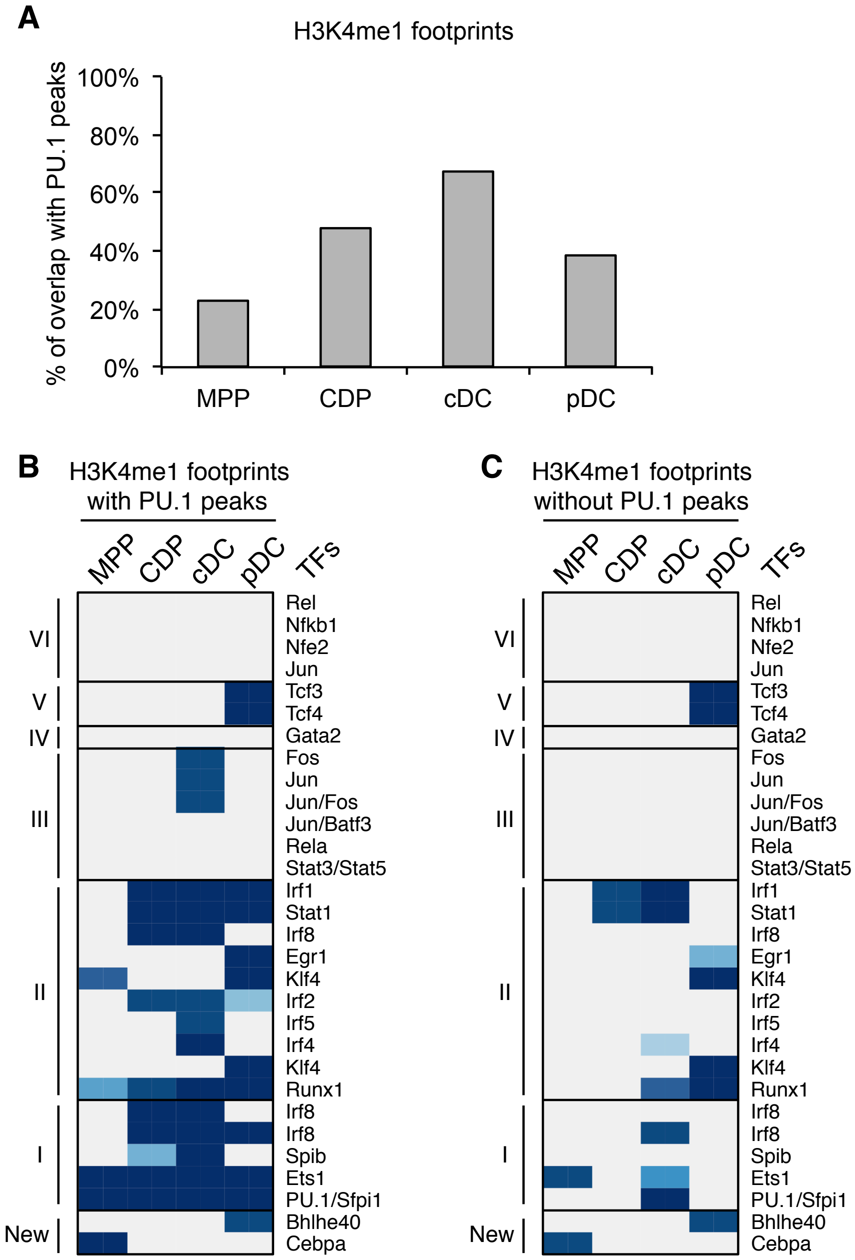

Supplementary Figure S8. Enrichment of transcription factor motifs for enhancer elements.
(A) The overlap of PU.1 peaks with H3K4me1 footprints, which represent the enhancer elements, during DC development. **(B)** Heat map depicts the enrichment of transcription factor motifs in MPP, CDP, cDC and pDC based on H3K4me1 footprints with PU.1 peaks. *P* values are plotted and color-coded using a continuous spectrum from gray (p value > 0.05) to blue (p value < 0.05). The genes were arranged according to their order in Figure 4A, with two newly identified transcription factors at bottom. **(C)** The enrichment analysis of transcription factor motifs based on H3K4me1 footprints without PU.1 peaks, which identified much less transcription factors.

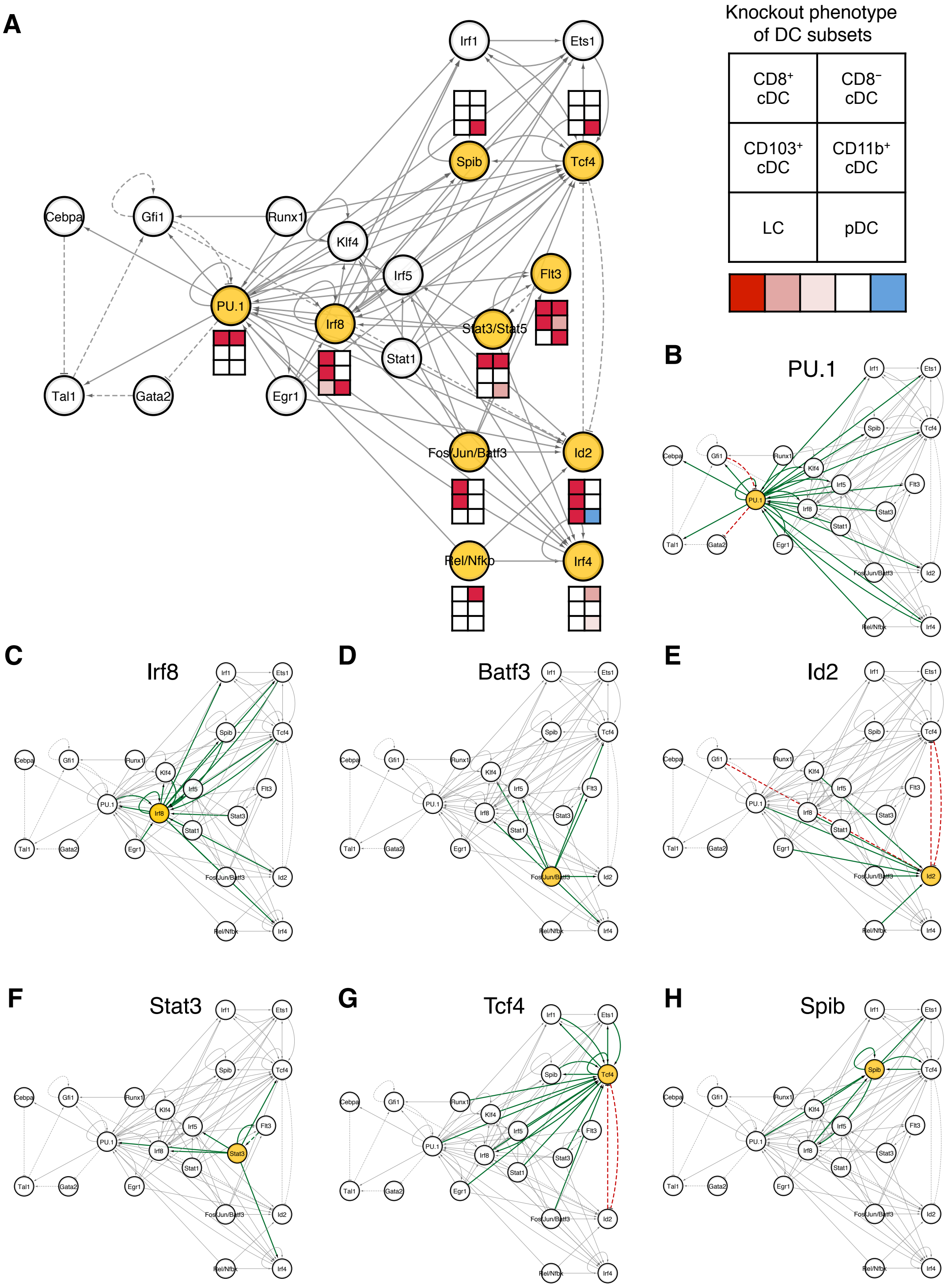

Supplementary Figure S9. Knockout phenotype of key genes in the regulatory circuitry of DC development.

(A) The knockout phenotype of PU.1, Irf8, Stat3/Stat5, Flt3, Batf3, Relb, Id2, Irf4, Spib and Tcf4 in multiple DC subsets (24,25) were shown in heatmap format and projected on the network. The color code indicates the impact of the factor on DC subsets (CD8^{+} cDC, CD8^{-} cDC, CD103^{+} cDC, CD11b^{+} cDC, LC and pDC) inferred from the respective knockout phenotype. Red, high impact, loss of respective DC subset in knockout mice; white, no impact or not determined; blue, gain of respective DC subset in knockout mice. (B-H) Connections of respective transcription factors (yellow) within the DC regulatory circuitry.

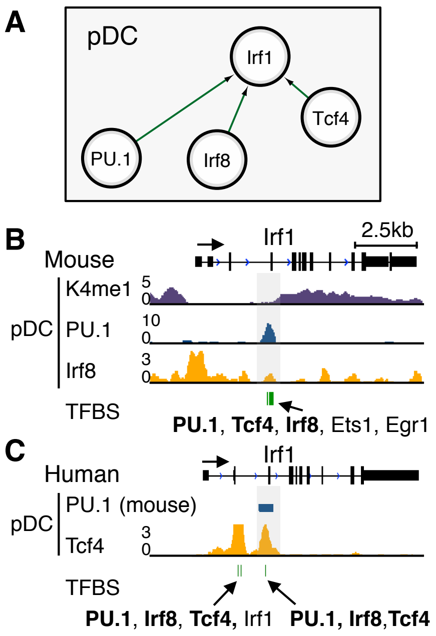

Supplementary Figure S10. Cobinding of PU.1, Irf8 and Tcf4 in Irf1 gene in pDC.
(A) PU.1, Irf8 and Tcf4 are predicted to impact on Irf1 in pDC (see network in Figure 6). **(B)** Occupancy of PU.1 and Irf8 in Irf1 enhancer region (H3K4me1) in pDC (highlighted in gray), which is in line with the predicted co-binding of Irf8 and PU.1. **(C)** The PU.1 peak in mouse pDC is mapped to human genome using UCSC liftOver tool and shown as blue bar. The PU.1 peak collocates with Tcf4 occupancy in human pDC and predicted transcription factor binding sites for PU.1, Irf8, Tcf4 and Irf1. The ChIP-seq data of Irf8 and Tcf4 in pDC are from GSE62702 and GSE43876. Predicted transcription factor binding sites (TFBS), green.

Supplementary References

1. Robinson, M.D., McCarthy, D.J. and Smyth, G.K. (2010) edgeR: a Bioconductor package for differential expression analysis of digital gene expression data. *Bioinformatics*, **26**, 139-140.
2. Benjamini, Y. and Hochberg, Y. (1995) Controlling the False Discovery Rate - a Practical and Powerful Approach to Multiple Testing. *Journal of the Royal Statistical Society Series B-Methodological*, **57**, 289-300.
3. Bailey, T.L., Boden, M., Whitington, T. and Machanick, P. (2010) The value of position-specific priors in motif discovery using MEME. *BMC Bioinformatics*, **11**, 179.
4. Portales-Casamar, E., Thongjuea, S., Kwon, A.T., Arenillas, D., Zhao, X., Valen, E., Yusuf, D., Lenhard, B., Wasserman, W.W. and Sandelin, A. (2010) JASPAR 2010: the greatly expanded open-access database of transcription factor binding profiles. *Nucleic Acids Res*, **38**, D105-110.
5. Robasky, K. and Bulyk, M.L. (2011) UniPROBE, update 2011: expanded content and search tools in the online database of protein-binding microarray data on protein-DNA interactions. *Nucleic Acids Res*, **39**, D124-128.
6. Heinz, S., Benner, C., Spann, N., Bertolino, E., Lin, Y.C., Laslo, P., Cheng, J.X., Murre, C., Singh, H. and Glass, C.K. (2010) Simple combinations of lineage-determining transcription factors prime cis-regulatory elements required for macrophage and B cell identities. *Mol Cell*, **38**, 576-589.
7. Marquis, J.F., Kapoustina, O., Langlais, D., Ruddy, R., Dufour, C.R., Kim, B.H., MacMicking, J.D., Giguere, V. and Gros, P. (2011) Interferon regulatory factor 8 regulates pathways for antigen presentation in myeloid cells and during tuberculosis. *PLoS Genet*, **7**, e1002097.
8. Kanno, Y., Levi, B.Z., Tamura, T. and Ozato, K. (2005) Immune cell-specific amplification of interferon signaling by the IRF-4/8-PU.1 complex. *J Interferon Cytokine Res*, **25**, 770-779.
9. Cock, P.J., Antao, T., Chang, J.T., Chapman, B.A., Cox, C.J., Dalke, A., Friedberg, I., Hamelryck, T., Kauff, F., Wilczynski, B. *et al.* (2009) Biopython: freely available Python tools for computational molecular biology and bioinformatics. *Bioinformatics*, **25**, 1422-1423.
10. Consortium, E.P. (2012) An integrated encyclopedia of DNA elements in the human genome. *Nature*, **489**, 57-74.
11. Ghosh, H.S., Cisse, B., Bunin, A., Lewis, K.L. and Reizis, B. (2010) Continuous expression of the transcription factor e2-2 maintains the cell fate of mature plasmacytoid dendritic cells. *Immunity*, **33**, 905-916.
12. Laiosa, C.V., Stadtfeld, M. and Graf, T. (2006) Determinants of lymphoid-myeloid lineage diversification. *Annu Rev Immunol*, **24**, 705-738.
13. Carotta, S., Dakic, A., D'Amico, A., Pang, S.H., Greig, K.T., Nutt, S.L. and Wu, L. (2010) The transcription factor PU.1 controls dendritic cell development and Flt3 cytokine receptor expression in a dose-dependent manner. *Immunity*, **32**, 628-641.
14. Dahl, R., Iyer, S.R., Owens, K.S., Cuylear, D.D. and Simon, M.C. (2007) The transcriptional repressor GFI-1 antagonizes PU.1 activity through protein-protein interaction. *J Biol Chem*, **282**, 6473-6483.
15. Gottgens, B., Nastos, A., Kinston, S., Piltz, S., Delabesse, E.C., Stanley, M., Sanchez, M.J., Ciau-Uitz, A., Patient, R. and Green, A.R. (2002) Establishing the transcriptional programme for blood: the SCL stem cell enhancer is regulated by a multiprotein complex containing Ets and GATA factors. *EMBO J*, **21**, 3039-3050.
16. Li, H., Ji, M., Klarmann, K.D. and Keller, J.R. (2010) Repression of Id2 expression by Gfi-1 is required for B-cell and myeloid development. *Blood*, **116**, 1060-1069.
17. Staber, P.B., Zhang, P., Ye, M., Welner, R.S., Nombela-Arrieta, C., Bach, C., Kerenyi, M., Bartholdy, B.A., Zhang, H., Alberich-Jorda, M. *et al.* (2013) Sustained PU.1 levels balance cell-cycle regulators to prevent exhaustion of adult hematopoietic stem cells. *Mol Cell*, **49**, 934-946.

18. Suh, H.C., Gooya, J., Renn, K., Friedman, A.D., Johnson, P.F. and Keller, J.R. (2006) C/EBPalpha determines hematopoietic cell fate in multipotential progenitor cells by inhibiting erythroid differentiation and inducing myeloid differentiation. *Blood*, **107**, 4308-4316.
19. Shannon, P., Markiel, A., Ozier, O., Baliga, N.S., Wang, J.T., Ramage, D., Amin, N., Schwikowski, B. and Ideker, T. (2003) Cytoscape: a software environment for integrated models of biomolecular interaction networks. *Genome Res*, **13**, 2498-2504.
20. Hon, G., Wang, W. and Ren, B. (2009) Discovery and annotation of functional chromatin signatures in the human genome. *PLoS Comput Biol*, **5**, e1000566.
21. Gusmao, E.G., Dieterich, C., Zenke, M. and Costa, I.G. (2014) Detection of active transcription factor binding sites with the combination of DNase hypersensitivity and histone modifications. *Bioinformatics*, **30**, 3143-3151.
22. Miller, J.C., Brown, B.D., Shay, T., Gautier, E.L., Jojic, V., Cohain, A., Pandey, G., Leboeuf, M., Elpek, K.G., Helft, J. *et al.* (2012) Deciphering the transcriptional network of the dendritic cell lineage. *Nat Immunol*, **13**, 888-899.
23. Garber, M., Yosef, N., Goren, A., Raychowdhury, R., Thielke, A., Guttman, M., Robinson, J., Minie, B., Chevrier, N., Itzhaki, Z. *et al.* (2012) A high-throughput chromatin immunoprecipitation approach reveals principles of dynamic gene regulation in mammals. *Mol Cell*, **47**, 810-822.
24. Belz, G.T. and Nutt, S.L. (2012) Transcriptional programming of the dendritic cell network. *Nat Rev Immunol*, **12**, 101-113.
25. Merad, M., Sathe, P., Helft, J., Miller, J. and Mortha, A. (2013) The dendritic cell lineage: ontogeny and function of dendritic cells and their subsets in the steady state and the inflamed setting. *Annu Rev Immunol*, **31**, 563-604.
